# Serum markers of cardiac complications in a systemic sclerosis cohort

**DOI:** 10.1038/s41598-022-08815-8

**Published:** 2022-03-18

**Authors:** Anders H. Tennøe, Klaus Murbræch, Henriette Didriksen, Thor Ueland, Vyacheslav Palchevskiy, Stephen S. Weigt, Håvard Fretheim, Øyvind Midtvedt, Torhild Garen, Cathrine Brunborg, Pål Aukrust, Øyvind Molberg, John A. Belperio, Anna-Maria Hoffmann-Vold

**Affiliations:** 1 Department of Rheumatology, Oslo University Hospital, Postbox 4959, 0424 Nydalen, Oslo Norway; 2 Institute of Clinical Medicine, University of Oslo, Oslo, Norway; 3 Department of Anesthesiology, Stavanger University Hospital, Stavanger, Norway; 4 Department of Cardiology, Oslo University Hospital, Oslo, Norway; 5 Section of Clinical Immunology and Infectious Diseases, Oslo University Hospital, Oslo, Norway; 6 Department of Medicine, David Geffen School of Medicine at UCLA, Los Angeles, CA USA; 7 Oslo Centre for Biostatistics and Epidemiology, Research Support Services, Oslo University Hospital, Oslo, Norway; 8 K.G. Jebsen Inflammatory Research Center, University of Oslo, Oslo, Norway; 9 Research Institute of Internal Medicine, Oslo University Hospital, Oslo, Norway

**Keywords:** Biomarkers, Cardiology, Rheumatology

## Abstract

Primary cardiac involvement is one of the leading causes of mortality in systemic sclerosis (SSc), but little is known regarding circulating biomarkers for cardiac SSc. Here, we aimed to investigate potential associations between cardiac SSc and candidate serum markers. Serum samples from patients of the Oslo University SSc cohort and 100 healthy controls were screened against two custom-made candidate marker panels containing molecules deemed relevant for cardiopulmonary and/or fibrotic diseases. Left (LV) and right ventricular (RV) dysfunction was assessed by protocol echocardiography, performed within three years from serum sampling. Patients suspected of pulmonary hypertension underwent right heart catheterization. Vital status at study end was available for all patients. Descriptive analyses, logistic and Cox regressions were conducted to assess associations between cardiac SSc and candidate serum markers. The 371 patients presented an average age of 57.2 (± 13.9) years. Female sex (84%) and limited cutaneous SSc (73%) were predominant. Association between LV diastolic dysfunction and tumor necrosis factor-related apoptosis-inducing ligand (TRAIL) (OR 0.41, 95% CI 0.21–0.78, p = 0.007) was identified. LV systolic dysfunction defined by global longitudinal strain was associated with angiopoietin 2 (ANGPT2) (OR 3.42, 95% CI 1.52–7.71, p = 0.003) and osteopontin (OPN) (OR 1.95, 95% CI 1.08–3.52, p = 0.026). RV systolic dysfunction, measured by tricuspid annular plane systolic excursion, was associated to markers of LV dysfunction (ANGPT2, OPN, and TRAIL) (OR 1.67, 95% CI 1.11–2.50, p = 0.014, OR 1.86, 95% CI 1.25–2.77, p = 0.002, OR 0.32, 95% CI 0.15–0.66, p = 0.002, respectively) and endostatin (OR 1.86, 95% CI 1.22–2.84, p = 0.004). In conclusion, ANGPT2, OPN and TRAIL seem to be circulating biomarkers associated with both LV and RV dysfunction in SSc.

## Introduction

Systemic sclerosis (SSc) is a multi-organ, progressive disorder hallmarked by dysregulation of the immune system, vasculopathy and target organ fibrosis^1–3^. The heart is frequently affected in SSc and is together with lung involvement reported to account for the majority of disease-related causes of deaths^4–7^. The pro-fibrotic pathophysiology in SSc is still inadequately understood^1^. Cardiac fibrosis in SSc is thought to be related to repeated focal ischaemia leading to irreversible lesions and/or inflammatory myocarditis, but these issues have not been fully elucidated^8,9^. From our recent studies and others, we know that left and right ventricular (LV and RV) systolic dysfunction, as well as LV diastolic dysfunction, are common complications of SSc^10–12^. LV diastolic dysfunction further progresses over time and displays a poor prognosis^10^. We have also presented multivariable models predicting mortality in SSc^10,11^. Such models may be improved by additional predictor variables as knowledge of SSc pathology improves. Early detection of these cardiac complications is believed beneficial to improve outcome^10,11^. However, there exist no established guidelines for screening of cardiac involvement as for pulmonary arterial hypertension (PAH) where screening with annual echocardiography is recommended^13,14^.

The structure–function data provided by echocardiography is highly useful to diagnose established cardiac dysfunctions. However, to identify SSc patients at risk of developing, or progression of cardiac dysfunction, there is a need for easily accessible algorithms including biological markers. The detection of SSc associated pulmonary hypertension (PH) is based on clinical, imaging, but also serological markers^15^. N-terminal pro-brain natriuretic peptide (NT-proBNP) is a highly valued serological marker of general cardiac dysfunction^16^. However, apart from the serum autoantibody anti-topoisomerase I (ATA) and NT-proBNP, there exist to our knowledge limited data on serological markers associated with cardiac dysfunction in SSc^17–19^.

Biomarkers are a prioritized research area in SSc as they may bring information regarding the disease itself and/or disease-related organ afflictions. Biomarkers may be divided into three categories: (i) diagnostic, (ii) prognostic for disease extent and severity and (iii) predictive for distinct organ complications and/or responsiveness to therapeutic intervention^20,21^. In SSc, we have previously shown that the homeostatic chemokine CCL21 and the anti-angiogenic molecule endostatin are associated with, and predict, new onset PAH^22–24^. Other studies have described additional markers with diagnostic and/or prognostic potential in other pro-fibrotic disorders with cardiopulmonary complications, justifying an evaluation in cardiac SSc. In example, the extracellular matrix protein osteopontin (OPN) is reported related to cardiac fibrosis in mice^25^ and tumor necrosis factor-related apoptosis-inducing ligand (TRAIL) has been associated with PH in humans and rodents^26,27^. Finally, as the interplay between fibrosis and immunological dysregulation in SSc is poorly understood, there is hope that investigation of associations between the disease and circulating serum markers linked to pro-fibrotic pathways may give clues to underlying pathophysiologic mechanisms and potentially novel treatment approaches.

In the present study, we assessed relations between two custom-made serum marker panels containing molecules deemed relevant for cardiac, pulmonary and/or fibrotic diseases and early stage SSc as a first proof of concept study. Biomarkers related to SSc were further evaluated for diagnostic and predictive abilities for cardiac dysfunction and mortality, respectively.

## Materials and methods

### SSc study cohort

All SSc patients seen at the department of rheumatology at Oslo University Hospital are included in the prospective Oslo SSc cohort, and are followed on an annual basis^22^. At first visit, patients undergo clinical examination (including assessment of digital ulcers, calcinosis and modified Rodnan skin score), serum sampling (including anti-centromere (ACA) and anti-topoisomerase-I (ATA) antibody status), pulmonary function tests, high-resolution CT (HRCT) scan and echocardiography^10^. Annual follow-up consultations are performed by a rheumatologist, registering data on demographic, clinical, laboratory, functional and imaging parameters in the Norwegian Systemic Connective Tissue Disease and Vasculitis Registry (NOSVAR)^22^. Ischemic heart disease (IHD) was defined presence of an International Classification of Diseases-10 (ICD-10) IHD-code from electronic patient journals (EPJs), while hypertension (HTN) was defined as a composite of an ICD-10 HTN-code *and* a systolic blood pressure > 140 mmHg at time of echocardiography.

This study included patients presenting at our hospital from 2002–2016. Inclusion criteria for this study were (i) fulfillment of the 1980 American College of Rheumatology criteria for SSc^28^ and/or the 2013 European League Against Rheumatism/American College of Rheumatology criteria^29^, (ii) age > 18 years and (iii) serum samples available for serum marker analysis. Demographic, clinical and laboratory data were extracted from NOSVAR and EPJs. Patients were classified as limited (lcSSc) or diffuse cutaneous (dcSSc) SSc according to skin involvement^30^. Data on medication were collected from EPJs and registered if ever used. Vital status was available for all patients at study end, extracted from the Norwegian national registry. Disease duration was defined as time from SSc diagnosis to serum sampling and observation period as time from serum sampling to death or study end. The study was approved by The Regional Committee of Health and Medical Research Ethics in South-East Norway, research protocols no. 2016/119 and 2017/1815. All included participants consented to serum sampling for research purposes. All methods were performed in accordance with the relevant guidelines and regulations.

### Serum sampling and biomarker analyses

Serum samples are collected from all SSc patients entering NOSVAR at first visit (baseline) and annual visits, using a standardized protocol for analyzation following the European Scleroderma Trial and Research (EUSTAR) recommendations for biobanking^31^. For this study, baseline sera were used. Serum was centrifuged within 30 min at room temperature and stored at − 70 °C before analyzation. Two custom-made panels were applied including specific circulating markers tailored for cardiac and pulmonary disease.Markers were identified through searches of PubMed with the search terms “biomarker, interstitial lung disease, cardiac disease, pulmonary diseases and systemic sclerosis”, in 2012 and 2015. After this search, expert opinion and author discussions led to the final markers. Samples from 2003–2016 were in 2016 analyzed by Luminex (panel A), using Milliplex assays (Merck Millipore), evaluating markers known from immune-related and pro-fibrotic disease states^32,33^. In 2013, serum samples collected from 2002–2013 were analyzed by enzyme-linked immunosorbent assay (ELISA) (panel B) (R&D Systems) for serum markers known to associate with cardiac or pulmonary disease^22,34^. All serological markers of Panel A and B are listed in Supplementary Table 1. One hundred randomly selected blood bank donors served as healthy controls, solely regarding serum marker sampling, and were analyzed in both steps. According to Norwegian law, only individuals without infection, cardiovascular disease, immunodeficiency or any chronic disease are allowed to donate blood^22^.

### Cardiopulmonary assessment

We had comprehensive cardiac assessments available. Systolic and diastolic cardiac function were evaluated by one of the authors (AHT) on available echocardiographies, applying updated international recommendations as previously shown^10,35^. Systolic dysfunction was defined by a global longitudinal strain (GLS) > − 17.0%^11,36^, rather than ejection fraction, due to better reproducibility. LV diastolic dysfunction was evaluated according to the 2016 guidelines as described earlier^10,37^ and RV systolic dysfunction was defined by tricuspid annular plane systolic excursion (TAPSE) < 17 mm^11,35^.

Suspicion of PH was based on patient history, symptoms/findings of PH, echocardiography findings, or the DETECT algorithm^15,38^, warranting referral to right heart catheterization (RHC) for PH diagnosis. According to recent suggestions from the 6th World Symposium for PH, precapillary PH was defined as mean pulmonary artery pressure (mPAP) ≥ 21 mm Hg, pulmonary arterial wedge pressure ≤ 15 mm Hg and pulmonary vascular resistance (PVR) ≥ 3.0 Wood units^39^. PAH and PH associated with ILD (PH-ILD) were defined as precapillary PH in absence or presence of ILD, respectively^38^. ILD was expressed as extent of ILD in percentage of total lung volumes on HRCT by semi-quantitative scoring method as previously described^39^. Briefly, area measurements were done precisely by drawing the region of interest to score the overall extent of fibrosis and relate this to the total lung volume. Fibrosis was expressed as the percentage of total lung volume. Significant ILD was defined as presence of ILD affecting > 10% of pulmonary tissue^5,40^. Extent of ILD on HRCT of 0.1–10% was defined mild ILD^5^.

### Outcome measures

As this was the first proof of concept study assessing circulating biomarkers in cardiac dysfunction in SSc, we aimed to first assess sera levels in SSc compared to healthy controls. In a second step, we tested associations between biomarkers and cardiac dysfunction. Lastly, we assessed biomarker predictive ability for all-cause mortality. Outcome measures were as follows:

1. Serum levels of the respective markers in SSc patients compared to values of healthy controls. Markers showing significant up- or downregulated median values in clinical SSc subsets, compared to controls, were further analyzed for association with cardiac dysfunction.
2. Association between serum biomarkers and the presence of (i) *LV systolic dysfunction*, assessed by GLS (ii) *LV diastolic dysfunction* assessed by e’, E/e’, left atrial volume index and tricuspid regurgitant velocity and (iii) *RV systolic dysfunction* assessed by tricuspid annular plane systolic excursion (TAPSE) were determined in all cases with conducted echocardiography within three years of blood sampling.
3. Serum markers showing association with echocardiographic dysfunction were tested for *mortality predictive* ability.

### Statistical analyses

Statistical analyses were performed using STATA version 16 (StataCorp LLC, College Station, Texas, USA) and SPSS version 25 (IBM, Armonk, New York, USA). Independent t-tests and chi square tests were applied for between-group comparisons as appropriate to assess differences in sera levels between SSc patients and healthy controls.

Candidate predictors for logistic and cox regression analyses were selected by expert opinion and based on the published literature^22,32–34^. Logistic regressions with odds ratios (OR) and 95% confidence intervals (CI) were applied for evaluation of association between cardiac outcomes and serological biomarkers. Parameters with significance levels < 0.20 were included in multivariable models adjusting for age and sex. Multivariable logistic regression analyses were preceded by correlation tests in order to avoid multicollinearity. In multivariable models, significance levels < 0.05 were considered significant. Multivariable models were evaluated by area under the receiver-operating curve (AUC), and models presenting values > 0.7 were considered acceptable and reported. Schoenfeld’s test was applied for affirmation of proportional hazards. For confirmation of clinical significance within the SSc cohort, serum markers associated with cardiac dysfunction were tested for mortality predicting abilities, adding them to multivariable models including diastolic dysfunction (model A) and precapillary PH (model B), as reported earlier^10^. These reported models included clinical-, imaging- and serological markers, and proved superior predictive ability compared to models with other appreciated risk factors, such as e.g. presence of IHD. Cox regressions with hazard ratios (HR) and 95% CI were calculated for the predictive value of serum biomarkers on mortality. C-statistics were applied to compare multivariable Cox models, with c-indexes > 0.7 representing acceptable models.

### Ethics approval and consent to participate

All patients provided consent to participate in the study. The study was approved by The Regional Committee of Health and Medical Research Ethics in South-East Norway.

## Results

### Study cohort and serum markers associating with SSc and selected disease features

**Figure 1:**
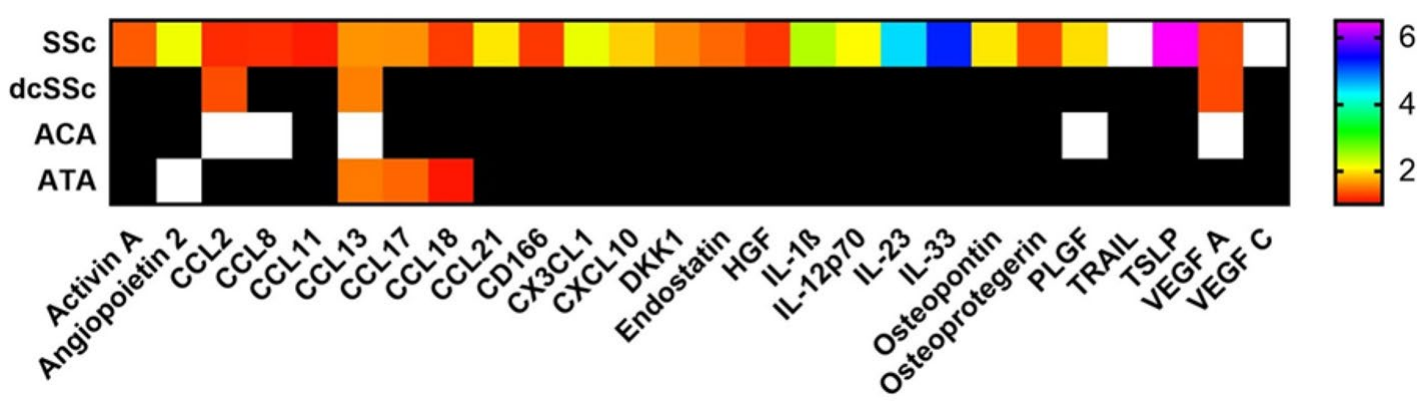
Heat map on circulating serum markers in SSc, diffuse SSc subset and SSc specific autoantibodies (Color print necessary). Mean serum marker values of the SSc group compared to healthy controls and reported as color ratios. Subsets are compared within the SSc cohort; dcSSc is compared to lcSSc, while ACA and ATA positive subsets are compared to ACA and ATA negative subsets, respectively. Colors reflect upregulated serum marker ratios, white squares indicate downregulation, and black squares indicate no significant alteration. *ACA* anti-centromere antibodies, *ATA* anti-topoisomerase-I antibodies, *CCL* C–C motif ligand, *CD* cluster of differentiation, *CXCL* C-X-C motif ligand, *dcSSc* diffuse cutaneous systemic sclerosis, *DKK1* Dickkopf-related protein 1, *HGF* hepatocyte growth factor, *IL* interleukin, *PLGF* placental growth factor, *SSc* systemic sclerosis, *TRAIL* tumor necrosis factor-related apoptosis-inducing ligand, *TSLP* thymic stromal lymphopoietin, *VEGF* vascular endothelial growth factor.

The study cohort included 371 SSc patients with serum samples that could be analyzed for markers of panel A containing immune-related molecules and other proteins linked to pro-fibrotic and inflammatory pathways. Among these, 255 patients (69%) had serum samples also analyzed for markers of panel B, enriched for molecules previously shown to associate with human cardiac or pulmonary diseases^41,42^. There were no differences between patient characteristics and demographics with or without panel B evaluation with respect to age, sex, subtype or autoantibodies. Most patients were female (n = 313, 84%) and presented with lcSSc (n = 269, 73%). Average age at serum sampling for panel A was 57 ± 14 years and median disease duration was 2.5 years (IQR 0.7–8.1). Demographic and serological features are shown in Table 1. Data on cardiovascular medications are shown in Supplementary Table 2. Seventy-four patients presented with precapillary PH (46 with PAH and 28 with PH-ILD). To assess the first outcome measure, serum levels of the individual panel A and B markers were compared between the SSc patients and healthy controls. Markers with significantly altered serum levels in SSc patients compared to controls are displayed in Fig. 1. We identified altered levels of 26 serum markers in SSc with varying levels in dcSSc, and the two major SSc specific autoantibodies, ACA and ATA (Fig. 1).

**Table 1:** Systemic sclerosis specific demographics and cardiac outcome measures of patients evaluated by panel A.

	Patients with available data, n (%)	Panel A
Age at serum sampling, years	364/371 (98)	57.2 (13.9)
Female, n (%)	371/371 (100)	313 (84)
Disease duration, years	364/371 (98)	2.5 (0.7–8.1)
Observation period, years	363/371 (98)	5.9 (3.1–8.3)
Mortality, n (%)	371/371 (100)	103/371 (28)
lcSSc, n (%)	362/371 (98)	269 (73)
ACA, n (%)	370/371 (99)	196 (53)
ATA, n (%)	370/371 (99)	58 (16)
mRSS	355/371 (96)	5 (2–13)
BMI, kg/m^2^	243/371 (65)	24 (4)
ILD, > 10% fibrosis	356/371 (96)	87 (24)
Ever smoking	234/371 (63)	135 (58)
Hypertension	246/371 (66)	31 (13)
Ischemic heart disease	246/371 (66)	42 (17)
GLS > -17.0%, n (%)	111/371 (30)	30 (27)
Diastolic dysfunction, n (%)	135/371 (36)	37 (27)
TAPSE < 17 mm, n (%)	167/371 (45)	29 (17)

Data are presented as mean (SD), median (IQR) or number (percentage).

*ACA* anti-centromere antibodies, *ATA* anti-topoisomerase I antibodies, *BMI* body mass index, *GLS* global longitudinal strain, *ILD* interstitial lung disease, *lcSSc* limited cutaneous systemic sclerosis, *mRSS* modified Rodnan skin score, *TAPSE* tricuspid annular plane systolic excursion. Parameters of cardiac function are evaluated on echocardiographies performed within three years from blood sampling.

### Patients evaluated for cardiac dysfunction in the study cohort

**Figure 2:**
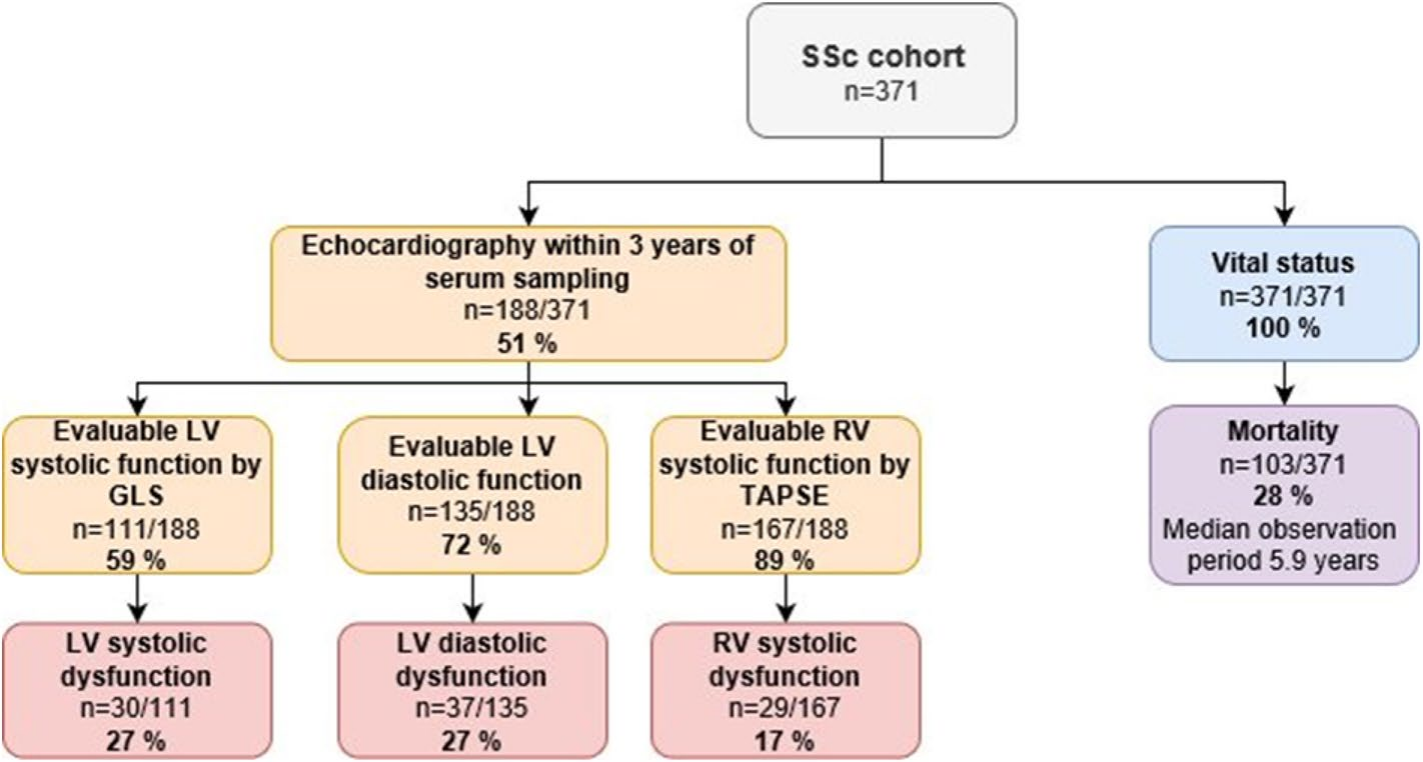
Flow chart on cardiac function and vital status of the SSc cohort. *GLS* global longitudinal strain, *LV* left ventricular, *RV* right ventricular, *SSc* systemic sclerosis, *TAPSE* tricuspid annular plane systolic excursion.

Echocardiographic data on GLS, LV diastolic dysfunction and/or TAPSE, within three years from serum sampling, was available in 188/371 (51%) study cohort patients (Fig. 2). Patients with available echocardiography within 3 years from serum sampling were older than the patients without available echocardiography (60.0 vs 54.2 years, p < 0.001), they had a shorter observation period (4.9 vs 7.5 years, p < 0.001) and higher mortality (38% vs 17%, p < 0.001). There were no differences with respect to sex, disease subtype, antibodies (ATA and ACA) or disease duration at serum sampling.

### Serum markers associated with LV systolic dysfunction

To assess the second outcome, association of circulating biomarkers and cardiac dysfunction including *LV systolic dysfunction*, *LV diastolic dysfunction* and *RV systolic dysfunction* in SSc, we analyzed all patients with available sera and echocardiographic data.

LV systolic dysfunction assessed by GLS was available in 111/188 (59%), of whom 30/111 (27%) had systolic dysfunction defined as GLS > − 17.0% (Fig. 2). Compared to patients with normal GLS, the patients with low GLS were more often male (43% vs 11%, p < 0.001), but did not differ regarding age, subtype, ACA, ATA, precapillary PH, disease duration or observation period. By adjusting for age and sex in logistic regression, systolic dysfunction by GLS showed significant association to two of the disease-linked serum markers displayed in Fig. 1; angiopoietin 2 (ANGPT2) and OPN (Table 2).

**Table 2:** Association between echocardiographic ventricular dysfunction and serum markers adjusted for age and sex.

Serum marker	OR	95% CI	p-value
**Left ventricular systolic dysfunction, GLS > -17%**			
ANGPT2 (n = 100)	3.42	1.52–7.71	0.003
OPN (n = 93)	1.95	1.08–3.52	0.026
**Left ventricular diastolic dysfunction**			
*ANGPT2 (n* = 122)*	*1.38*	*0.98–1.94*	0.062*
*Endostatin (n* = 113)*	*1.60*	*0.95–2.67*	0.075*
TRAIL (n = 131)	0.41	0.21–0.78	0.007
**Right ventricular systolic dysfunction, TAPSE < 17 mm**			
ANGPT2 (n = 153)	1.67	1.11–2.50	0.014
Endostatin (n = 143)	1.86	1.22–2.84	0.004
OPN (n = 143)	1.86	1.25–2.77	0.002
TRAIL (n = 163)	0.32	0.15–0.66	0.002

*ANGPT2* angiopoietin 2, *CI* confidence interval, *GLS* global longitudinal strain, *OPN* osteopontin, *OR* odds ratio, *TAPSE* tricuspid annular plane systolic excursion, *TRAIL* tumor necrosis factor-related apoptosis-inducing ligand.

*Indicates numerical associations. HR of ANGPT2, Endostatin, OPN and TRAIL represent an increase of one standard deviation.

### Serum markers associated with left ventricular diastolic dysfunction

Echocardiographic evaluation of LV diastolic dysfunction by e’, E/e’, left atrial volume index and tricuspid regurgitant velocity according to recommendations, was performed within three years of serum sampling in 135 of the study cohort patients with available echo (72%). Among these, 37/135 (27%) presented with diastolic dysfunction. There were no differences between patients segregated by diastolic function with respect to disease duration, sex, SSc subtype, ACA or ATA. Patients with diastolic dysfunction had higher age (64.6 vs 55.8 years, p < 0.001), more frequent coexisting precapillary PH (67% vs 21%, p < 0.001) and shorter observation period (2.6 vs 6.1 years, p < 0.001) than patients with normal diastolic function. By adjusting for age and sex in logistic regression, diastolic dysfunction was associated with TRAIL, while showing numerical associations with ANGPT2 and endostatin (Table 2).

### Serum markers associated with RV systolic dysfunction

RV systolic dysfunction was assessed by TAPSE in 167/188 patients (89%), with TAPSE < 17 mm, indicating RV systolic dysfunction, present in 29/167 patients (17%). Patients with low TAPSE presented numerical higher age (63.1 vs 58.3 years, p = 0.074), more frequent precapillary PH during the observation period (64% vs 30%, p = 0.001) and shorter observation period (2.8 vs 5.3 years, p < 0.001) than patients with normal TAPSE. There were no differences between patients segregated by low/normal TAPSE with respect to disease duration, sex, SSc subtype, ACA or ATA. In multivariable logistic regression adjusting for age and sex, RV systolic dysfunction was independently associated with ANGPT2, endostatin, OPN and TRAIL.

Sera levels of ANGPT2, OPN and TRAIL of SSc patients compared to healthy controls are shown in Supplementary Fig. 1. Sub-group analyses of serum marker levels were performed on patients with echocardiography < 6 months from serum sampling. This group showed, as SSc patients in general, highly significantly altered levels of OPN (n = 85), TRAIL (n = 111) and ANGPT-2 (n = 111), compared to controls (p < 0.001 for all three serum markers). However, this group included too few patients with outcomes of cardiac dysfunction to perform multivariable analyses.

### Serum markers associated with mortality

Over the observation period of median 5.9 years (IQR 3.1–8.3), 103/371 patients (28%) deceased. Patients who died were older (65.5 vs 53.9 years, p < 0.001), had longer disease duration at serum sampling (3.9 vs 2.0 years, p = 0.004), shorter observation period (2.6 vs 7.5 years, p < 0.001), were more often male (22.3% vs 13.1%, p = 0.028), and presented more often dcSSc (34.3% vs 22.3%, p = 0.019), compared to survivors.

In our previous publication, we have presented two multivariable prediction models for SSc mortality^10^. When we added serological markers separately to Model A (including diastolic dysfunction, sex, age at echocardiography, RV systolic function, diffusion capacity of the lungs, NT-proBNP and extent of skin affliction), ANGPT-2 and OPN remained independent predictors of mortality in their respective models (Table 3), yet excluding NT-proBNP from Model A due to insignificant p-value. When we added cardiac markers separately to Model B (including precapillary PH, sex, age at echocardiography, NT-proBNP and extent of skin affliction), ANGPT-2 and TRAIL remained independent predictors of mortality, retaining significance for all parameters of the original model (Table 4).

**Table 3:** Prediction of mortality by angiopoietin 2 and OPN in multivariable cox regression.

Multivariable cox regression on mortality							
	HR	95% CI	p-value		HR	95% CI	p-value
ANGPT2	1.52	1.20–1.93	0.001	OPN	1.39	1.01–1.92	0.044
Age	1.04	1.01–1.08	0.024	Age	1.05	1.01–1.09	0.023
Diastolic dysfunction	3.71	1.62–8.48	0.002	Diastolic dysfunction	3.73	1.76–7.90	0.001
DLCO, %	0.95	0.93–0.97	< 0.001	DLCO, %	0.95	0.93–0.97	< 0.001
Male sex	0.94	0.37–2.36	0.888	Male sex	0.79	0.30–2.11	0.638
mRSS	1.07	1.03–1.11	< 0.001	mRSS	1.05	1.01–1.08	0.006
TAPSE	0.32	0.13–0.83	0.018	TAPSE	0.26	0.12–0.57	0.001
C-index	0.87			C-index	0.89		

*ANGPT2* angiopoietin 2, *CI* confidence interval, *DLCO* diffusion capacity of the lungs for carbon monoxide, *HR* hazard ratio, *mRSS* modified Rodnan skin score, *OPN* osteopontin, *TAPSE* tricuspid annular plane systolic excursion. HR of ANGPT2 and OPN represent an increase of one standard deviation.

**Table 4:** Prediction of mortality by ANGPT2 and TRAIL in multivariable cox regression.

Multivariable cox regression on mortality							
	HR	95% CI	p-value		HR	95% CI	p-value
ANGPT2	1.33	1.07–1.67	0.012	TRAIL	0.66	0.44–0.99	0.045
Age	1.07	1.04–1.10	< 0.001	Age	1.07	1.04–1.09	< 0.001
Male sex	1.18	0.63–2.22	0.598	Male sex	1.23	0.67–2.25	0.510
mRSS	1.05	1.02–1.08	< 0.001	mRSS	1.04	1.01–1.06	0.005
NT-proBNP	1.01	1.01–1.01	< 0.001	NT-proBNP	1.01	1.01–1.01	< 0.001
Precapillary PH	2.54	1.44–4.48	0.001	Precapillary PH	2.03	1.19–3.46	0.009
C-index	0.83			C-index	0.82		

*ANGPT2* angiopoietin 2, *CI* confidence interval, *HR* hazard ratio, *mRSS* modified Rodnan skin score, *NT-proBNP* N-terminal prohormone of brain natriuretic peptide, *PH* pulmonary hypertension, *TRAIL* tumor necrosis factor-related apoptosis-inducing ligand. The HR of ANGPT2 and TRAIL represent an increase of one standard deviation.

### Serum markers and relation to pulmonary disease

By logistic regression, neither OPN nor TRAIL showed association to ILD. OPN and TRAIL showed no correlation with mPAP, PVR, cardiac output (CO) nor cardiac index (CI). Angiopoietin showed a numerical association with ILD (p = 0.16) and a weak correlation with mPAP, PVR and CI, but not with CO.

## Discussion

LV and RV systolic and diastolic dysfunction are frequent cardiac complications of SSc and are associated with substantial reduced survival. Today, cardiac involvement often remains subclinical until severe organ manifestation is evident, and is often diagnosed at an advanced disease stage. Biomarkers revealing patients at high risk of cardiac dysfunction and increased mortality are scarce to date and therefore in demand^10,11^.

In the present study, we investigated candidate serum markers from two custom-tailored large panels for cardiopulmonary disease as a proof of concept study. We identified several markers associated with SSc in general, and several associated with cardiac disease. Our findings show an independent association of ANGPT2 with LV and RV systolic dysfunction, and mortality strongly linking this molecule to cardiac dysfunction in SSc. In addition, OPN, endostatin and TRAIL were associated with cardiac dysfunction, and notably, TRAIL was associated with diastolic dysfunction as well as mortality.

ANGPT2, OPN and TRAIL have all been previously shown altered in SSc. In addition, they have been reported altered in general cardiac disease^41,43,44^. These markers were therefore of particular interest for evaluation of cardiac disease associated with SSc. To our knowledge, this is the first study to present data on the association between biomarkers and cardiac dysfunction in SSc, potentially also representing novel targets for therapy in these patients. Here we show strong associations between these three circulating markers with LV and/or RV systolic dysfunction, and for TRAIL also with LV diastolic dysfunction. The three markers were also independently associated to mortality. In addition to prognostic and predictive potential, a greater understanding of these serum markers’ physiologic characteristics may provide insight into the insufficiently comprehended pathophysiology of SSc. We do not expect these markers to replace the well appreciated NT-proBNP, and we have therefore neither fully investigated their association to NT-proBNP. However, this work is considered to be hypothesis generating, with a potential to enlighten and expand our knowledge on cardiac SSc. Given the retrospective nature of this study, it was not possible to relate the aforementioned markers to clinical cardiac symptoms.

ANGPT2 is produced by endothelial cells^45^ and presumed to impair angiogenesis and promote cardiac fibrosis^43^. ANGPT2 is an antagonist of ANGPT1, an anti-inflammatory protein stimulating vascular integrity and homeostasis^45^. Our study supports earlier reports on higher serum concentrations of ANGPT2 in SSc patients in general, but without assessment to cardiac dysfunction^46^. ANGPT2 has been suggested a potential therapeutic target against pathologic angiogenesis and inflammation^45^. It is unclear whether ANGPT2 contributes to SSc pathophysiology or whether increased levels may reflect cardiac tissue at distress, or both. Future studies on whether ANGPT2 may predict cardiac disease, and whether regulation of ANGPT2 expression may have a therapeutic potential, are in demand.

TRAIL is a cytokine inducing endothelial cell apoptosis and smooth muscle proliferation^27^. Serum levels are shown reduced in rheumatoid arthritis patients with heart failure^47^. Among patients with acute coronary syndrome, low TRAIL concentration is reported a strong predictor of poor prognosis^44^. Further, it is suggested that inhibition of TRAIL may reverse PH ^27^. While our cohort presented reduced levels of TRAIL in cardiac SSc, an earlier small scale study (n = 30) reported *increased* serum values of TRAIL among SSc patients compared to healthy controls, with no relation to cardiac involvement^48^. Different results may also reflect dual effects of apoptosis. Thus, while apoptosis could attenuate inflammation at an early stage of disease, it could enhance cardiac failure in a fibrotic myocardium.

OPN is a cytokine recruiting macrophages and T-cells to site of inflammation. In rodents, OPN is reported upregulated in cardiac fibrosis^25^ and to promote collagen synthesis^49^, possibly at least at an early stage protecting the LV from dilation and systolic dysfunction. OPN has further been suggested as a therapeutic target^41^. These findings are in concordance with our data, showing higher values in patients with reduced LV and/or RV systolic function. One may speculate whether regulation of OPN may reduce the burden of cardiac and/or pulmonary fibrosis in SSc. Future studies on the therapeutic potential of OPN regulation are in demand.

ANGPT2, OPN and TRAIL were all also strong predictors of mortality in our SSc cohort; even when adjusting for general risk factors of SSc. NT-proBNP lost significance when either ANGPT2 or OPN were included in multivariable model A including age, sex, diffusion capacity of the lungs, modified Rodnan skin score, NT-proBNP, LV diastolic dysfunction and RV systolic function. NT-proBNP is a highly recognized serum marker of heart failure, extensively utilized in clinical cardiology. NT-proBNP levels increase both with elevated cardiac pressures and reduced renal filtration. This may suggest a cardiac, or possibly renal, impact of ANGPT2 and OPN. As these two markers excluded NT-proBNP from prediction Model A, one may speculate whether these markers reveal cardiac, or non-cardiac, relations to mortality, superior to the ability of NT-proBNP. The roles of ANGPT2 and OPN are therefore especially interesting and call for future research approaches. The focus of this study is however not to design a prediction model for primary cardiac affection from SSc, but to cast light on novel promising serum markers.

Echocardiographic findings of cardiac dysfunction may be due to primary cardiac affliction, non-SSc general cardiac disease or PH, the most feared complication of SSc. Increased pulmonary pressures increase RV pressure and may cause septal shift towards the LV, impairing LV filling during diastole. While the reported associations may reflect primary cardiac dysfunction due to SS, we cannot disregard that some of these associations may alternatively reflect precapillary PH. However, in this report, we show ANGPT2 and TRAIL to be independent predictors of mortality, adjusted for precapillary PH in multivariable analyses. Further, severe diastolic dysfunction is a very common cause of PH in the general population. Reports indicate that a considerable amount of patients with PH due to diastolic dysfunction may be misclassified as precapillary PH^50,51^, enlightening some of the association between diastolic dysfunction and precapillary PH. It is therefore of vital importance to evaluate the impact of cardiac SSc on mortality. We were not able to compare levels of the aforementioned serum markers between pre- and postcapillary PH due to a low number of patients with postcapillary PH, which did not allow for meaningful statistical analyses.

Our study possesses some limitations worth recognizing. SSc patients may present cardiac dysfunction from non-SSc etiologies, which may dilute the characteristics of SSc-related cardiac dysfunction. Second, as echocardiographies were recorded from 2003, novel parameters as GLS were only evaluable for half of the cohort. Third, the nature of our study did not allow simultaneous serum sampling and echocardiography. In order to assure a temporal relation, only patients with a maximum of three years between echocardiography and sampling were included in analyses on GLS, diastolic dysfunction and TAPSE. Fourth, patients and controls were not completely matched to age and sex, which might have had an effect on serum marker levels. Last, while we consider our well-examined cohort favorable for evaluation of biomarker associations, we lack a validation cohort and validation studies are in demand.

The study also possesses significant strengths. This proof of concept serum marker study is to our knowledge the first study on serum markers of echocardiographic verified cardiac dysfunction in SSc. The study includes data on multiple parameters of a well-characterized cohort. Both panels were custom tailored for specific circulating markers known from cardiopulmonary disease. The levels were compared to a large control group of healthy individuals, analyzed at the time of patient serum analysis. Third, while the rarity of echocardiographic abnormalities limits the number of outcomes for analyzation purposes, the large size of our cohort allowed us to evaluate independent associations between serum markers and cardiac dysfunction using multivariable regression analyses.

## Conclusion

In this study, we have shown ANGPT2, endostatin, OPN and TRAIL to be altered in SSc patients with cardiac dysfunction. ANGPT2, OPN and TRAIL were further strong independent predictors of mortality in combination with known risk factors for mortality in SSc. These markers may help enlighten the inadequately understood pathomechanisms of cardiac SSc, and could even potentially prove valuable for the diagnosis and treatment of this detrimental disease.

## Supplementary Information

Supplementary Information.

## Data Availability

The datasets used during the current study are available from the corresponding author on reasonable request.

## Abbreviations

ANGPT2: Angiopoietin 2
GLS: Global longitudinal strain
PAH: Pulmonary arterial hypertension
PH-ILD: Pulmonary hypertension due to interstitial lung disease
OPN: Osteopontin
TAPSE: Tricuspid annular plane systolic excursion
TRAIL: Tumor necrosis factor-related apoptosis-inducing ligand
