# Supplementary material for: Serum markers of cardiac complications in a systemic sclerosis cohort
Source: Sci Rep. 2022 Mar 18;12:4661. doi: 10.1038/s41598-022-08815-8 (PMC8933514; doi:10.1038/s41598-022-08815-8)
Supplement: Supplementary file 1 — Supplementary Information. [file 41598_2022_8815_MOESM1_ESM.docx]

**Data Supplements:**

Supplementary Table 1: Serological markers evaluated by Panel A and B.

| **Panel A** | **Panel B** |
| --- | --- |
| Angiopoietin 2 | Activin A |
| CCL2 | CCL17 |
| CCL3 | CCL18 |
| CCL4 | CCL19 |
| CCL5 | CCL21 |
| CCL8 | CD166 antigen |
| CCL11 | CX_3_CL1 |
| CCL13 | CXCL10 |
| CCL22 | DKK1 |
| CCL24 | Endostatin |
| CXCL13 | Lipocalin-2 |
| Fibroblast growth factor 1 | Osteoprotegerin |
| Fibroblast growth factor 2 | Osteopontin |
| Hepatocyte growth factor | Pentraxin-related protein PTX3 |
| IL-1 receptor antagonist |  |
| IL-1β |  |
| IL-6 |  |
| IL-8 |  |
| IL-10 |  |
| IL-12p70 |  |
| IL-17a |  |
| IL 23 |  |
| IL 33 |  |
| Placental growth factor |  |
| Thymic stromal lymphopoietin |  |
| Tumor necrosis factor-related apoptosis-inducing ligand |  |
| VEGF A |  |
| VEGF C |  |
| VEGF D |  |

Supplementary Table 2: Additional clinical features and medication of SSc patients evaluated by panel A.

|  | Patients with available data,  n (%) | Panel A |
| --- | --- | --- |
| Digital ulcers, n (%) | 361/371 (97) | 155 (43) |
| Calcinosis, % | 281/371 (76) | 122/281 (43) |
| Any pulmonary fibrosis, n (%) | 356/371 (96) | 198/356 (56) |
| Severe pulmonary fibrosis > 10 %, n (%) | 356/371 (96) | 87/356 (24) |
| PAH, n (%) | 371/371 (100) | 46 (12) |
| PH-ILD, n (%) | 371/371 (100) | 28 (8) |
| Acetylsalicylic acid, n (%) | 246/371 (66) | 101/246 (41) |
| Beta blockers, n (%) | 246/371 (66) | 81/246 (33) |
| Calcium channel blockers, n (%) | 246/371 (66) | 195/246 (79) |
| ACE-I/ARB II, n (%) | 246/371 (66) | 99/246 (40) |
| Diuretics, n (%) | 246/371 (66) | 105/246 (43) |
| Statins, n (%) | 245/371 (66) | 99/245 (40) |
| Anticoagulants, n (%) | 246/371 (66) | 67/246 (27) |

ACE-I, angiotensin-converting-enzyme inhibitor; ARB II, angiotensin II receptor blocker; PAH, pulmonary arterial hypertension; PH-ILD, pulmonary hypertension from interstitial lung disease. PAH/PH-ILD data are described to be complete as even patients at low suspicion of PH are referred for right heart catheterization.

Supplementary Table 3: Systemic sclerosis specific demographics and cardiac outcome measures of patients evaluated by panel B.

|  | Patients with available data,  n (%) | Panel B |
| --- | --- | --- |
| Age at serum sampling, years | 298/298 (100) | 56.0 (13.8) |
| Female, n (%) | 298/298 (100) | 249 (84) |
| Disease duration, years | 285/298 (96) | 2.5 (0.5-8.7) |
| Observation period, years | 298/298 (100) | 7.9 (5.3-9.5) |
| Mortality, n (%) | 298/298 (100) | 104 (35) |
| lcSSc, n (%) | 295/298 (99) | 222 (75) |
| ACA, n (%) | 298/298 (100) | 151 (51) |
| ATA, n (%) | 298/298 (100) | 50 (17) |
| mRSS | 275/298 (92) | 6 (3-13) |
| ILD | 290/298 (97) | 75 (26) |
| BMI, kg/m^2^ | 210/298 (70) | 24 (4) |
| Ever smoking | 200/298 (67) | 122 (61) |
| Hypertension | 213/298 (71) | 25 (12) |
| Ischemic heart disease | 213/298 (71) | 39 (18) |
| GLS > -17.0 %, n (%) | 93/298 (32) | 25 (27) |
| Diastolic dysfunction, n (%) | 113/298 (38) | 28 (25) |
| TAPSE < 17 mm, n (%) | 143/298 (48) | 21 (15) |

Data are presented as mean (SD), median (IQR) or number (percentage). ACA, anti-centromere antibodies; ATA, anti-topoisomerase I antibodies; BMI, body mass index; GLS, global longitudinal strain; lcSSc, limited cutaneous systemic sclerosis; mRSS, modified Rodnan skin score; TAPSE, tricuspid annular plane systolic excursion. Parameters of cardiac function are evaluated on echocardiographies performed within three years from blood sampling.

Supplementary Figure 1: Serum levels of angiopoietin 2, osteopontin and TRAIL in SSc patients and healthy controls
